# A Droplet Microfluidic Sensor for Point-of-Care Measurement of Plasma/Serum Total Free Thiol Concentrations

**DOI:** 10.1021/acs.analchem.4c04163

**Published:** 2025-01-27

**Authors:** Liam Carter, Adrian Nightingale, Martin Feelisch, Xize Niu

**Affiliations:** 1 Mechanical Engineering, Faculty of Engineering and Physical Sciences, University of Southampton, Southampton SO17 1BJ, U.K.; 2 Perioperative and Critical Care Theme, NIHR Southampton Biomedical Research Centre, University Hospital Southampton, Southampton SO16 6YD, U.K.; 3 Clinical & Experimental Sciences, Faculty of Medicine, University of Southampton, Southampton SO17 1BJ, U.K.

## Abstract

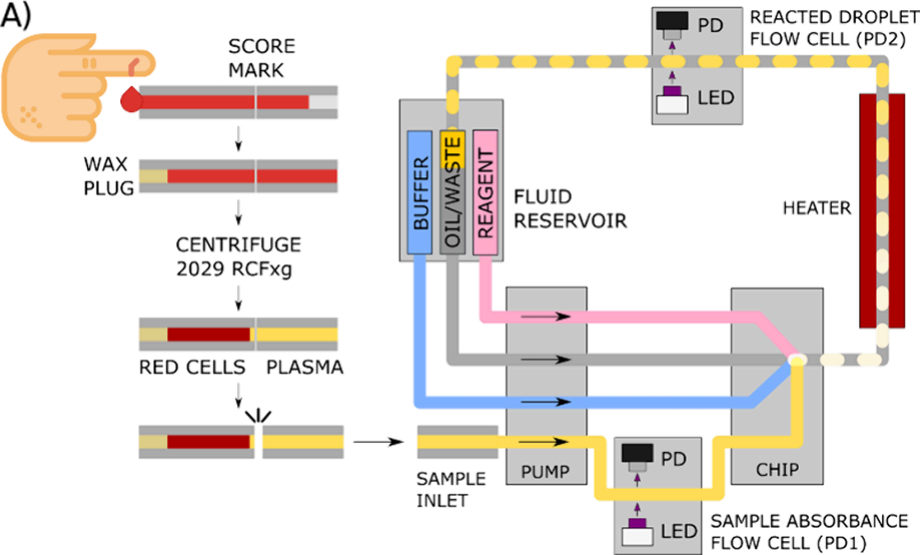

Total free thiols are an important marker of the whole-body redox state, which has been shown to be associated with clinical outcome in health and disease. Recent investigations have suggested that increased insight may be gained by monitoring alterations of redox state in response to exercise and hypoxia and to monitor redox trajectories in disease settings. However, conducting such studies is challenging due to the requirement for repeated venous blood sampling and intensive lab work. Droplet microfluidic sensors offer an alternative platform for developing a point-of-care testing approach using small sample volumes and automated systems to complement or ultimately replace laboratory testing. Here we developed a small, portable droplet microfluidic sensor that can measure total free thiol concentrations in 20 μL human plasma (or serum) samples, providing a reading in less than 10 min. This system features a novel method to enhance the mixing of reagent and analyte in droplets containing viscous biological fluids. The results in a range of real-world human plasma samples showed equivalence with current standard laboratory assays while reducing sample volume requirements 9-fold and fully automating the process. Micro hematocrit capillaries allowed testing of capillary blood samples collected by fingerprick lancing. The system was used to monitor total free thiols using fingerprick samples in healthy volunteers and revealed significant changes in total free thiols in response to food intake and exercise. This device has the potential to improve our ability to conduct physiological studies of total free thiol level changes and improve our understanding of redox physiology, which may ultimately be applied in redox medicine to improve patient care.

Within the human body, numerous redox couples work in concert to support homeostasis and maintain redox balance, and measurements in extracellular fluids such as blood plasma can provide useful insight into the overall `redox state’.^1−5^ Oxidative stress occurs when an oxidizing load is applied to the system that shifts the balance between the formation of oxidizing species and the antioxidant defense mounted toward oxidation.^6^ Minor oxidative loads such as short bouts of exercise are managed using redox buffering capacity, in the form of antioxidant enzymes and molecules which are sacrificially oxidized to prevent cell/tissue damage. Chronic exposure to moderate levels of oxidative stress leads to increased buffering capacity^7^ and may contribute to the favorable association between enhanced physical activity and reduced risk of diabetes, cancer, and cardiovascular disease.^8,9^ However, excessive oxidative stress can overwhelm the endogenous buffering capacity, damaging cell membranes, DNA, and other molecular constituents.^10^ Oxidative stress is thought to be a significant risk factor in numerous disease settings ranging from diabetes and neurodegenerative and cardiovascular diseases to sepsis, cancer, and COVID-19.^11−15^ Single time-point measurements of redox markers have been correlated with increased cardiovascular mortality in the general population^16^ and clinical outcome in patients with coronary artery disease.^17^ Currently, measurements of the redox environment and levels of oxidative stress experienced are generally limited to research studies using laboratory analyses not routinely available to clinicians.

Thiols (molecules containing a sulfhydryl (-SH) group) are not merely markers of intracellular or extracellular redox state but themselves involved in redox regulation by acting as “redox switches” that alter the structure and function of proteins such as transcription factors, enzymes, and ion channels, responding to a changing environment and functioning on multiple levels in the oxidant defense mechanism.^5^ Thiols are characterized by their oxidation state and either classified as “free thiols” when in their reduced state ready to act as an antioxidant or in the form of “disulfides” when the molecule has been oxidized^18^ (the latter can exist in free form or bound to a protein). Free thiols are commonly measured spectrophotometrically using Ellman’s reagent (5,5′-dithiobis-2-nitrobenzoic acid, DTNB), containing a weak disulfide (S–S) bond which is reduced by SH groups to form a mixed disulfide and the yellow-colored product 2-nitro-5-thiobenzoic acid (TNB) with a peak absorbance at 412 nm.^19^ As multiple molecules containing free thiol groups will participate in this reaction, the assay provides a measure of total-free-thiols (TFT). In plasma or serum, the single free SH group of albumin accounts for the majority of the TFT signal, with minor contributions of reduced glutathione and other free low-molecular weight thiols. Measurement of TFT in serum and plasma has been correlated with the severity of multiple diseases such as autoimmune disease lupus nephritis,^20^ COVID-19 (where TFT provides an independent risk factor for disease severity),^21^ and the survival of both patient and graft after renal transplantation,^22^ among other conditions including myocardial infarction,^23^ heart failure,^12^ and sepsis.^11,24^ Importantly, TFTs have been demonstrated to behave dynamically in response to oxidative stress, decreasing in response to exercise, with the magnitude of the change correlated with the intensity of the exercise,^25^ and increasing on reperfusion of tissues after cold-induced vasoconstriction in healthy volunteers and patients suffering from Raynaud’s disease.^26^ These studies indicate the potential for circulating thiols to be a dynamic biomarker that can track the state of the redox regulatory system. They also indicate the need for the facilitation of easier, faster testing of redox markers to begin monitoring changes in response to disease progression. Continuous or rapid point-of-care (POC) monitoring can fill this gap and facilitate a greater number of stimuli to be measured, contributing to a better understanding of the redox regulatory system.

Recent years have seen an increasing emphasis on personalized’ or “precision” medicine, where care is adjusted to an individual based on objectively quantifiable parameters. This trend has further highlighted the need for POC testing, particularly for devices which can monitor at frequent intervals or continuously.^27,28^ Monitoring of patient parameters facilitates the detection of trends or patterns, enabling the early identification of deteriorating conditions, which is critical for managing diseases such as sepsis. In particular, the analysis of trends in redox markers, such as TFTs, could be used to monitor disease progression or response to treatment in diseases associated with oxidative or reductive stress.

While the analysis of redox markers at the point of care is relatively new, there are a few examples. The RedoxSYS system (Luoxis Diagnostics) measures the overall redox state of plasma by electrochemically detecting the oxidation reduction potential (ORP) using a disposable electrode chip; using this system, statistically significant differences were observed in blood plasma ORP between survivors and nonsurvivors of pediatric cardiac surgery^29^ and in patients with and without sepsis.^30^ Two other commercial devices, the Callegari 1930-Form Plus 3000 and the EuroMedix-FRAS4 Evolvo,^31,32^ use colorimetric assays to measure antioxidant content in plasma. However, their operation requires significant manual handling, including pipetting, limiting their practical use and true POC nature, which has prevented their use in regular practice. A POC device that could perform standard assays, regularly or continuously, with minimal manual intervention would reduce barriers to adoption in research or clinical practice and increase the chances of successful incorporation into routine clinical care.

Our previous work involved developing a wearable droplet microfluidic platform,^33^ which demonstrated the capability to carry out spectrophotometric assays in nanolitre droplets, significantly reducing sample volume requirements. In this paper, we present the development of a fully integrated, portable, point-of-care sensor using droplet microfluidics for measuring total-free-thiol concentrations from fingerprick blood samples. Droplet microfluidics was selected over alternative methods, such as continuous-flow analysis, due to its rapid mixing within droplets and reduced risk of surface contamination (low carryover) in the microchannels. The system utilizes a phased peristaltic micropump, which enables a robust and compact design. This approach is compatible with a wide range of sampling methods, supporting both intermittent sampling as demonstrated here and potential future adaptations for continuous sampling methods like microdialysis with minimal modifications to the sensor.

## Methods

### Reagents and Materials

Cysteine, ethylenediamine tetraacetic acid (disodium salt dihydrate; EDTA-Na_2_), Tris-HCl, dibasic and monobasic sodium phosphate, 5,5′-dithio-bis(2-nitrobenzoic acid) (DTNB), and bovine serum albumin (BSA) were purchased from Merck and used without further purification. Ultrapure water (18.2 MΩ·cm, Milli-Q (MQ)) was used to prepare all aqueous solutions. Phosphate buffer (0.1 M) was prepared from sodium phosphate dibasic and sodium phosphate monobasic and adjusted to pH 7.0. Tris buffer (0.1 M) with 10 mM EDTA was prepared from Tris-HCL and 0.5 M EDTA and adjusted to pH 8.2. DTNB reagent was made up in concentrations from 1.9 to 10 mM in phosphate buffer and stored in opaque bottles in a fridge, to minimize degradation.^34^ Cysteine and BSA standards were made up in Tris buffer, snap-frozen in liquid nitrogen, stored at −80 °C and defrosted immediately before each experiment. The carrier oil used in all droplet microfluidic experiments was FC-40 (Fluorinert, 3M). A triblock polymer surfactant PFPE–PEG-PFPE was synthesized using a previously reported method^35,36^ and added to FC40 at 0.1% weight per volume where noted.

### Design of the Droplet Microfluidic System

**Figure 1:**
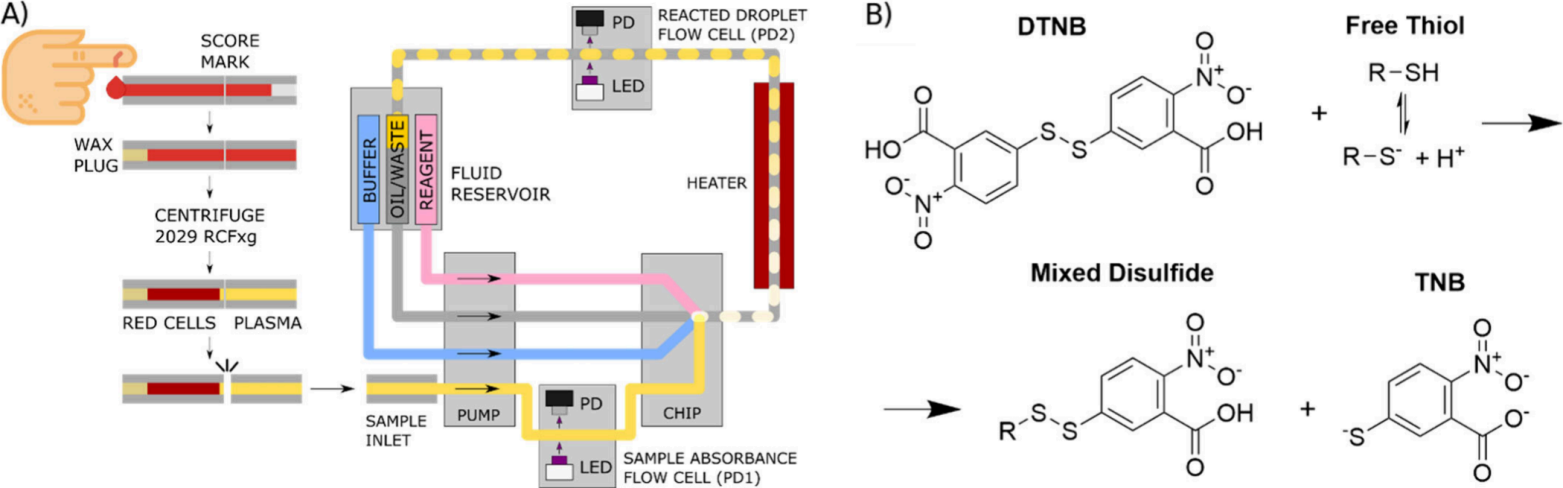
(A) Device schematic showing sample collection and processing in micro hematocrit capillaries followed by loading into the droplet microfluidic sensor for automated analysis. (B) Reaction of Ellman’s reagent (DTNB) with free thiols (RSH). Note that the entity reacting with the disulfide moiety in DTNB is the thiolate anion (RS^–^).

The droplet microfluidic system schematic is illustrated in Figure 1a. Fluids are controlled within the microfluidic system by a phased peristaltic style pump, based on our previous work,^37^ delivering precisely controlled volumes of sample, reagent, buffer and oil, in antiphase to produce droplets, as shown in Figure 1a. The geometry of the pump roller (the part that drives fluid through the pump tubing) was designed to give fluid dose volume ratios that matched those used in standard laboratory analysis, with the volume per droplet being approximately 1/100 of those used per well in the plate reader assay. The sample was first pumped through UT6 PTFE tubing (ID:0.6 mm, Adtech Polymer Engineering Ltd., U.K.) into a temperature controlled (37 °C) 3D-printed optical flow cell with a 390–425 nm LED (SLLP-F586-1520-UV-395, TSLC, RS) and light-to-voltage converter (TSL257, Farnell), labeled PD, to measure the background absorbance^38^ of the sample. All fluids were directed into a 3D-printed microfluidic chip (Elegoo gray ABS-like resin, Mars 3 Pro) with droplets generated into UT6 PTFE tubing inserted at the droplet generation point. UT6 tubing containing droplets was guided over a custom heating element (37 °C) by a 3D printed tubing guide, controlling the reaction temperature and time (1 min), and into a second temperature-controlled optical flow cell measuring the absorbance of the droplets post reaction. The background absorbance measured in the first flow cell was scaled to an equivalent absorbance, as would be measured by the second flow cell, which measured the reacted droplets. This was accomplished by using the experimentally measured dilution calibration curve as shown in Supporting Information Figure S3. This calibration accounts for the effective dilution of the input sample from the buffer and reagent in the microfluidic chip and any variation in optical performance between the two flow cells. The same background dilution calibration curve was used for all samples analyzed, where the scaled background sample absorbance was subtracted from the total absorbance in the second flow cell to give an isolated reaction absorbance. This background-corrected reaction absorbance can then be converted into a total-free thiol concentration using a calibration curve generated from cysteine standards processed by the same microfluidic system. The whole microfluidic system was packaged into a portable box (*L* × *W* × *H* = 160 mm × 120 mm × 90 mm) with integrated control electronics (Teensy 4.1), 12 V power, and serial data communication, as shown in Figure S1. Reagent, buffer, and oil were loaded into the pump via AWG30 PTFE tubing connected to a fluid reservoir. Samples were loaded via a 3D printed inlet adapter using either a piece of AWG30 PTFE tubing inserted into an Eppendorf vial or a 20 μL capillary.

### Blood Sampling

Blood samples were donated by healthy volunteers. This study was approved by the local Research Ethics Committee of the University of Southampton (ERGO, 68007; IRAS, 307839). Blood samples were collected via venepuncture into BD Vacutainer K_3_EDTA tubes. EDTA was selected as anticoagulant due to its transition metal ion chelation which acts to prevent oxidation of thiols during sample preparation.^39−41^ Plasma was collected by pipetting whole blood into 1.5 mL centrifuge tubes (Eppendorf) and centrifuging for 10 min in a Technico mini centrifuge (Fischer, U.K.) at a relative centrifugal force (RCF) 2000*g*, as per standard protocols.^42,43^ Plasma supernatant was pipetted off and used immediately or stored at −80 °C.

For capillary blood collection, Unistik 3 Extra 21Gx2mm (Owen Mumford) automatic lancets were used. Standard practice for the puncture was followed:^44^ before blood collection, the participant’s hand was warmed in a heating pad for 5 min, vasodilating the capillaries, to further increase blood flow within the hand and the participant was asked to windmill their arm several times. The participant’s fingerpad was cleaned using a 70% alcohol swab. The alcohol was allowed to evaporate, and the lancet was applied to the side of the distal segment of the finger. The lancet was activated, and the capillary blood was collected into 20 μL of heparin coated capillaries (Hirschmann, Minicaps). Where possible, blood was collected using passive flow through the puncture; however, in cases with limited blood flow the finger was massaged to encourage flow.

Before use, capillaries were scored around their outer diameter, 18 mm from the filling end, using a diamond-tipped scribe fixed in a 3D-printed jig. The filling end of the capillary was sealed with Hawksley Cristaseal wax and placed into a JOAN LAB MC-7Pro centrifuge (wax seal outward) and centrifuged for 10 min at 2029 RCFxg. To access plasma after separation, capillaries were cleanly snapped at the score mark located at the end of the plasma region, and the segment containing plasma was connected to the sample inlet with a 3D-printed adapter. This process is shown in the left part of Figure 1A.

### Laboratory Analysis

Adaptation of the traditional Ellman’s reagent assay, to improve its suitability for miniaturization and transfer into a rapid POC-style microfluidic system was conducted using standard laboratory analysis techniques with a FLUOstar Omega plate reader and 96 well plates, with each measurement repeated in triplicate wells. Performance of the microfluidic system was also benchmarked against these standard techniques. As with the microfluidic system, the sample background absorbance was measured prereagent addition and subtracted from the reacted absorbance measurement. For each well 22.5 μL of sample was diluted with 67.5 μL of Tris buffer, the absorbance measured, and then 50 μL of 10 mM DTNB was added and incubated at 37 °C for 1 min with the reacted absorbance recorded. For all samples, absorbance measurements were converted to TFT concentrations using the previously generated cysteine calibration curve.

## Results and Discussion

### Assay Miniaturization: Optimizing Reaction Conditions

TFTs can be measured using absorbance spectrophotometry and Ellman’s reagent (5,5′-dithiobis-2-nitrobenzoic acid, DTNB). The reagent contains a disulfide (S–S) bond which can be reduced quantitatively by free thiol (-SH) groups to form a mixed disulfide and the yellow-colored nitroaromatic product 2-nitro-5-thio-benzoic acid (TNB) with a peak absorbance at 412 nm,^19,34,39,45,46^ as shown in Figure 1B. The assay completes within 1 min at room temperature with low-molecular-weight (LMW) thiols such as cysteine. However, the reaction with high-molecular-weight (HMW) such as protein thiols, which represent the bulk of the signal in plasma, requires 5–30 min for completion, depending on reaction conditions.^40,47−49^ While protein thiols account for the majority of the TFT signal in plasma samples, their use for calibration is problematic. Potential HMW standards such as human or bovine serum albumin (HSA, BSA), which have a single free thiol group per molecule that is not engaged in forming a structural disulfide bond, but only a fraction of those remain both accessible for reaction and in the reduced state.^40,49,50^ In reference material, this fraction can vary dependent on preparation, conditions and duration of storage, and other factors, prohibiting its use as a calibration standard (in real-life samples, determination of the fraction of this particular SH-group is, of course, the whole point of performing the assay). It is therefore standard practice to use the LMW thiol cysteine, which contains one free SH-group per molecule and is relatively stable for calibration purposes. However, HMW thiols such as BSA do provide a surrogate to test the response to samples more representative of human plasma in which TFT is predominantly protein-bound.

**Figure 2:**
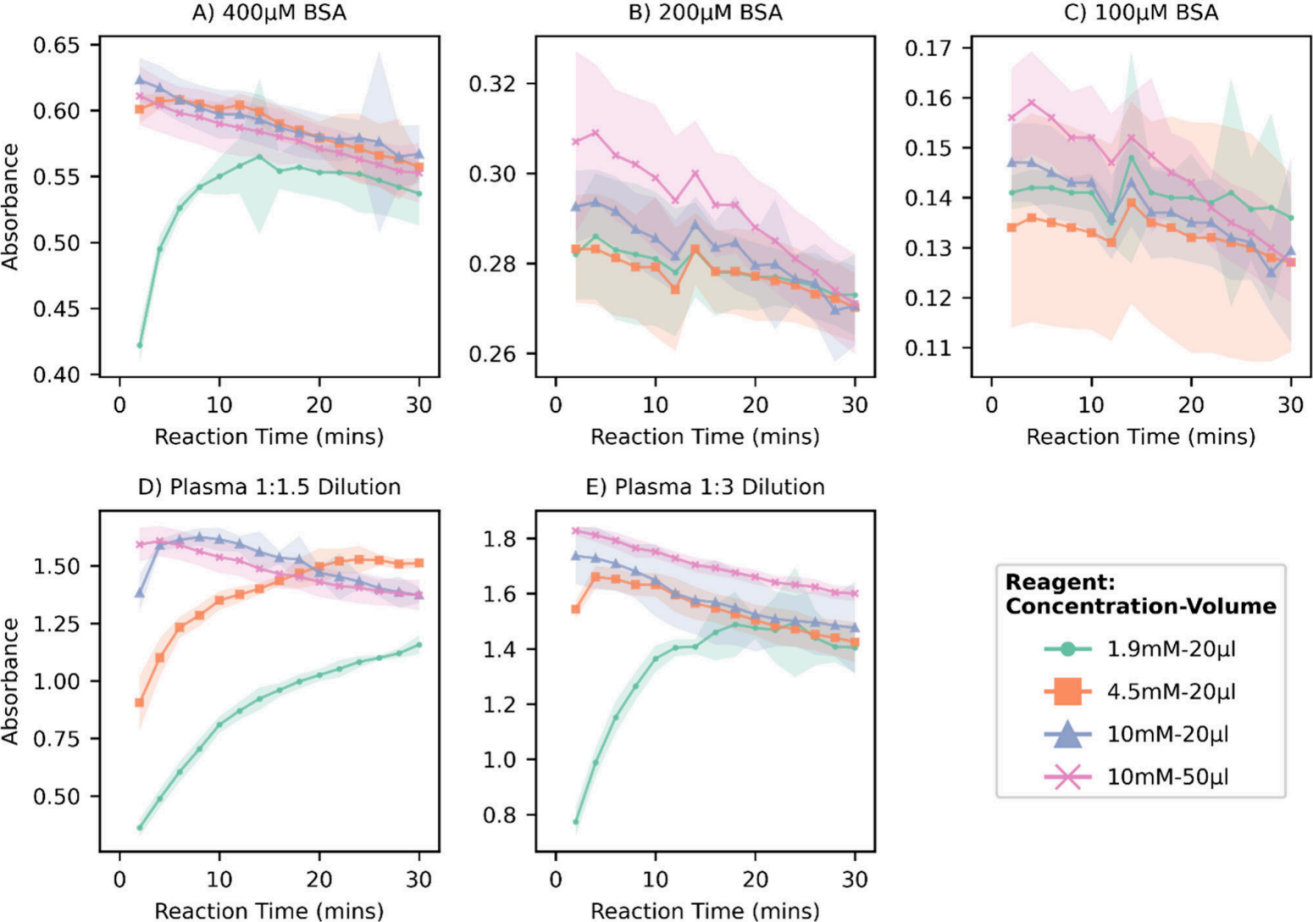
Plots of measured absorbance versus reaction times for varying reagent concentrations and sample volumes indicated in the legend: (A) 400 μM bovine serum albumin (BSA), (B) 200 μM BSA, (C) 100 μM BSA, (D) plasma diluted 1:1.5 with Tris buffer, (E) plasma diluted 1:3 with Tris buffer. Shaded regions show ±SD across 3 wells.

A POC-style device requires the sample loading-to-reading time to be as short as possible. Therefore, we first explored how different reaction parameters affected the reaction time of Ellman’s reagent with BSA an exemplary protein thiol, with key results shown in Figure 2. Various DTNB reagent concentrations and volumes as well as sample dilutions were tested to minimize the reaction time required without increasing reaction temperature or changing pH beyond physiological limits (since the thiolate rather than the protonated thiol reacts with the disulfide moiety (Figure 1B), the reaction is dependent not only on the p*K*_a_ of the SH group but also on reaction pH). The reaction rate was observed using a plate reader thermostated at 37 °C, for BSA solutions along with a plasma sample prepared at two dilutions in Tris buffer (1:1.5 and 1:3 plasma:buffer by volume). Cysteine calibrations were conducted in parallel to convert observed absorbances into TFT concentrations. A temperature of 37 °C was selected to reduce the reaction time while not exceeding physiological temperatures which may result in protein denaturation with exposure of SH groups not accessible physiologically, giving rise to false-positive readings.

For lower BSA concentrations, no difference was seen in the reagent configurations tested, with all samples showing maximal absorbance to be reached within 1 min. This was followed by a gradual decline in concentration due to the TNB product reacting via thiol-exchange with mixed disulfides, either formed from the initial reaction or present in the original samples.^39,40,51^ At higher BSA concentration (400 μM) and at both dilutions, variation in the time to peak was observed in the different reagent configurations, with increasing concentration and/or volume or reagent resulting in faster maximal absorbance, a behavior previously observed in the lower reagent concentration range of 0.1–1 mM.^49^ It was decided to proceed with 50 μL of 10 mM DTNB configuration for the remainder of the testing as this provided the most consistent and rapid reaction completion time of less than 1 min, across a range of samples and minimizes the chance of underestimating TFT concentration due to incomplete reaction. The microfluidic heater tubing path was set to provide a residence time of 1 min and the temperature set at 37 °C in all remaining testing, replicating the conditions in the plate reader.

### Microfluidic Calibration

With redox-sensitive assays such as that described here, it is not feasible to use a spiked recovery method for validation. In complex biological samples, such as plasma, a wide range of redox active molecules and electron exchange reactions exist in equilibrium. Addition of a reactive spiking compound, such as a thiol, will disrupt this equilibrium, leading to thiol–disulfide interchange reactions and a new unpredictable balance. This makes it challenging to accurately determine the resulting free thiol concentration. Consequently, external calibrations are necessary to validate the system.

**Figure 3:**
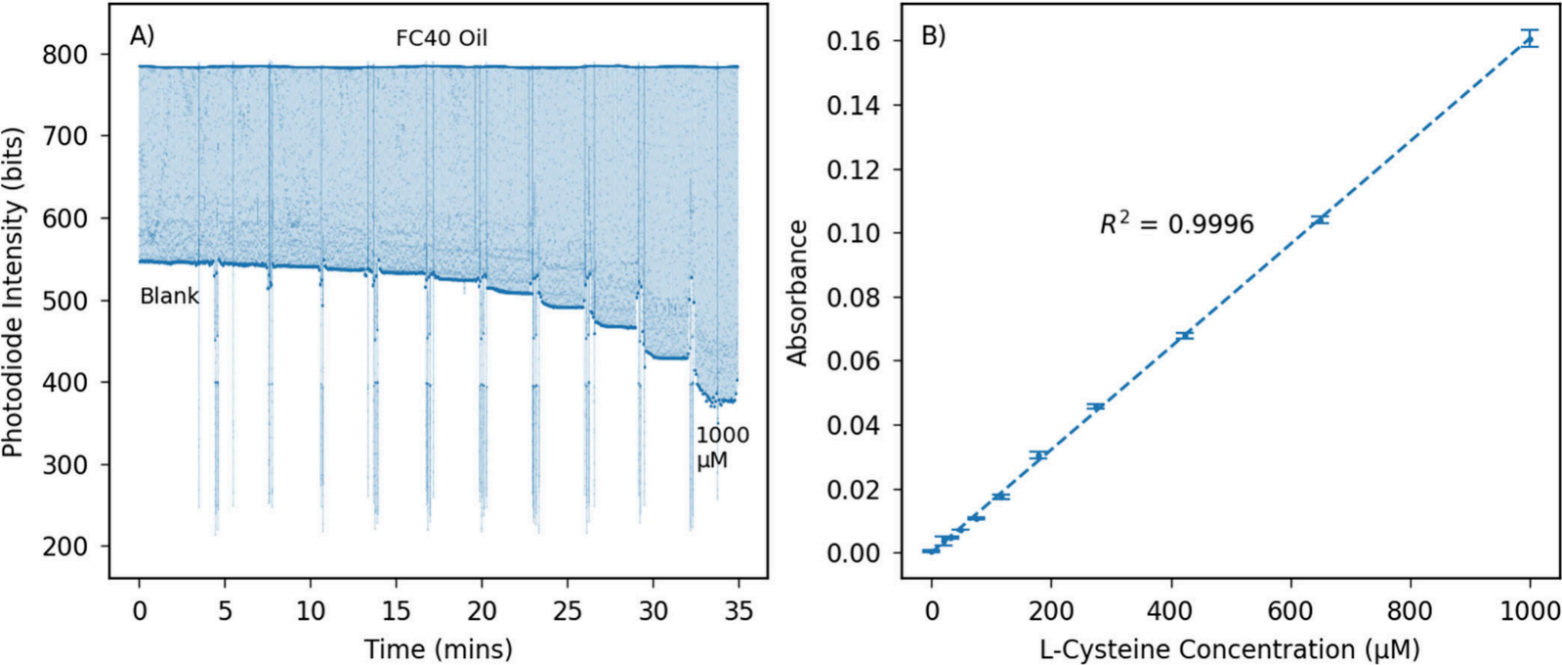
(A) Raw photodiode intensity data recorded during a cysteine calibration of the microfluidic system. FC40 region is identified along with the aqueous droplets containing blanks and 1000 μM cysteine; between these regions concentration of standard increases (20.7, 31.9, 49, 75.4, 116, 179, 275, 423, 650 μM) with a short air gap separating each standard. (B) Concentration–response curve for TFT microfluidic sensor using l-cysteine as standard; error bars show ±SD of 35 droplets.

The microfluidic sensor was calibrated using cysteine standards by loading the standard samples directly into the pump inlet with air drawn in between standards for clear differentiation. Figure 3A shows the raw light intensity data recorded by flow cell 2 monitoring the reacted droplets with a clear signal differential between aqueous droplets (low signal) and FC40 carrier oil (high signal). The absorbance of the droplets was calculated using a modified version of the Beer–Lambert law, including terms for the light transmission through the carrier oil preceding an aqueous droplet that allows for the correction of variable LED light intensity.^52^ A total of 45 droplets from each region were processed, with the first 10 discarded due to carryover, to generate a calibration curve, Figure 3B, showing a linear response up to at least 1,000 μM and a limit of detection of 1.7 μM measured using the 3 sigma definition.^53^ Despite the short optical path length (0.6 mm), this compares well with the LOD reported in the literature for traditional analysis (2.8–3.1 μM) and also as determined using our 96-well plate reader, 2.95 μM, indicating that the microfluidic system provides very stable reaction conditions. The system has a suitable dynamic range to measure TFT in plasma, previously reported to be in the range of 200–600 μM.^54,55^

### Microfluidic Measurement of Protein Bound Thiols

**Figure 4:**
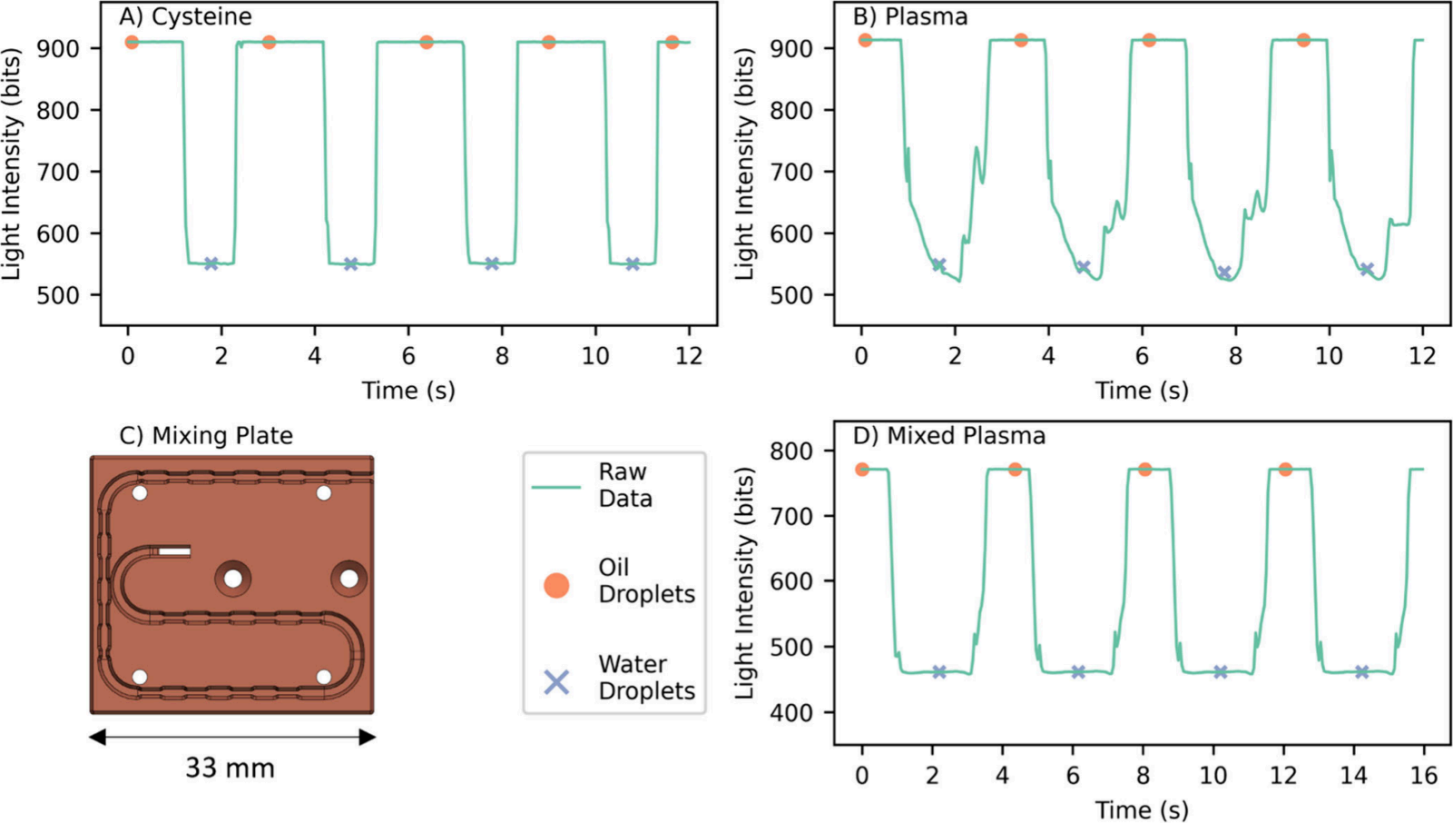
Raw photodiode intensity data and identified oil region and aqueous droplet means for droplets containing (A) 800 μM cysteine and (B) plasma traveling in regular PTFE tubing. (C) Construction of the 3D-printed bumpy mixing plate to intentionally introduce irregularities into the PTFE tubing path to enhance mixing. (D) Intensity data for droplets containing plasma traveling in bumpy constructed PTFE tubing with surfactant. Legend applies to parts A, B, and D.

Measurement of cysteine standards showed a clear distinction in the measured intensity of FC40 and aqueous droplets, with a consistent intensity recorded over the length of the aqueous droplets, Figure 4A. However, initial testing with BSA solutions or plasma as a sample showed significant deviation from the clean square wave intensity trace observed with aqueous cysteine solutions. In both cases, the intensity of the FC40 oil regions showed a consistent level with clean transitions; however, the aqueous region showed significant variability along the length of a droplet (Figure 4B), suggesting an inhomogeneous distribution within the droplet due to incomplete mixing. Droplet microfluidic systems typically show rapid mixing due to the action of recirculation flows within the droplet;^56−58^ however proteins, including BSA, have previously been shown to impede mixing within droplets as they absorb onto the droplet-oil interface^59^ leading to the generation of Marangoni stresses which oppose the internal recirculation flow. To improve the mixing of biological solutions containing proteins, we therefore introduced constrictions to serpentine microfluidic channels to generate greater shear stresses on the droplet interface, perturbing the Marangoni stresses and increasing the recirculation flows and mixing within the droplet.^59−61^ Additionally, surfactants have been shown to modify the behavior of proteins at the droplet boundary,^62^ potentially reducing Marangoni stresses and also improving mixing. Both of these strategies were therefore implemented in the microfluidic system. Constrictions were introduced into the UT6 PTFE tubing by adding bump features to a 3D-printed tubing guide which the tubing was pressed into (Figure 4C). A triblock polymer surfactant PFPE-PEG-PFPE, was synthesized^35,36^ and added to the FC40 at a loading of 0.1% weight. This surfactant has shown biocompatibility, prevention of protein adsorption to the surface boundaries, and excellent emulsion stabilization. The addition of both channel bumps and the surfactant resulted in a significant improvement in droplet mixing and resulting flow cell intensity signal for protein containing droplets (Figure 4D), offering a method to achieve improved mixing of complex biological solutions in PTFE channels.

**Figure 5:**
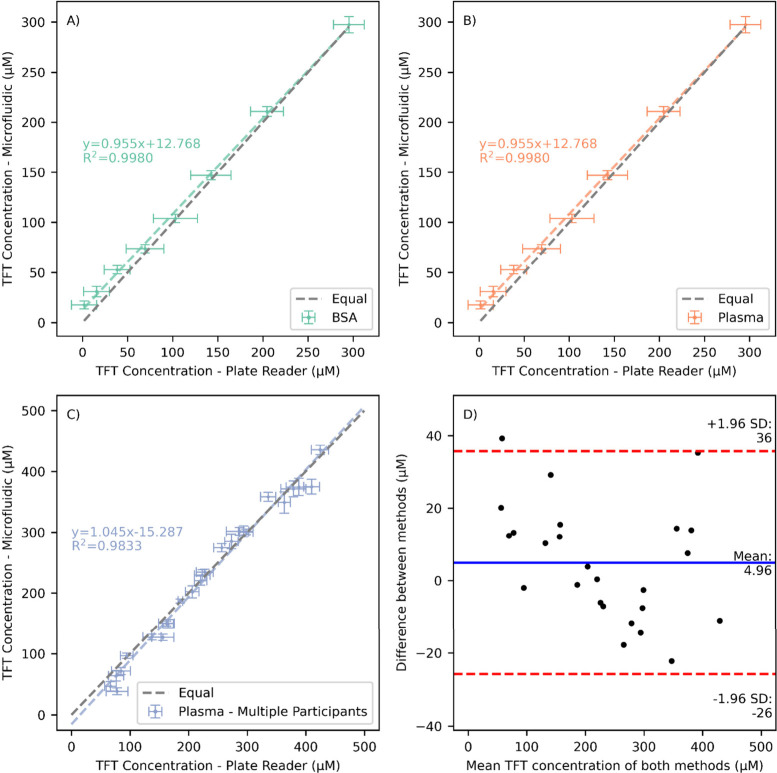
Comparison of microfluidic system and well plate based measurement of TFT concentrations for (A) serial dilutions of a 1000 μM BSA sample, (B) serial dilutions of a single participant’s plasma sample, (C) serial dilutions of 5 participants plasma samples. (D) Bland–Altman plot of the data in part C, showing mean difference between methods and 95% confidence intervals. Error bars show ±SD across 3 wells or 30 droplets for the well plate and microfluidic methods, respectively.

The microfluidic system with a bumpy mixing plate and surfactant was benchmarked against the plate reader with increasingly complex biological solutions. Initially, BSA solutions between 20.7 and 1000 μM were prepared and analyzed using the same method described for the cysteine calibration. The samples were parallel processed in triplicate wells in the plate reader for reference. Comparing directly with the well plate reader results showed a good correlation between the two systems, with the microfluidic system showing a lower standard deviation at each concentration than the well plate repeats (Figure 5A).

To test the system response in an authentic biological sample of relevance, a participant donated a plasma sample which was serially diluted by a factor of 0.7 a total of 7 times, testing over a range of TFT and protein content. Again, samples were processed in parallel for comparison with the plate-reader-based assay system. These plasma samples show even closer correlation between the systems than the BSA samples (Figure 5B), with a similar reduction in measured standard deviation in the microfluidic system. However, given that there is a greater standard deviation between droplets when measuring low concentration plasma samples, it may be more representative to use a low concentration plasma samples standard deviation in the calculation which gives a limit of detection of 12.8 μM, still well below the range of concentrations typically reported physiologically.

A further test was conducted on a set of plasma samples collected from five different participants, each sample was diluted 4 times giving five dilution levels for each original sample (neat, 80, 60, 40 and 20%) and a total of 25 samples for comparison with plate reader analysis. Again, a good correlation was found across the whole concentration range, typically with lower standard deviations for the microfluidic system (Figure 5C). The mean difference across all 25 samples is 4.95 μM lower for the microfluidic system than the well plate analysis, showing minimal bias, with a 95% confidence interval between the methods of −26 to +36 μM (Figure 5D); given the standard deviation for one sample can be in the range of 20 μM, this suggests that the two systems are performing equivalently and could be used interchangeably. This microfluidic sensor is therefore capable of measuring TFT concentration in real-world plasma samples with equivalence to current standard lab practice, while using lower sample volumes (10 μL vs 90 μL), with no manual processing steps required post plasma collection and a rapid sample-to-read time of less than 10 min, in a robust portable system.

### Minimizing Blood Volume

While the plasma sample volume required has been reduced, the initial whole blood sample still requires collection via venepuncture and processing of collected blood in a standard centrifuge. To facilitate sample collection, fingerprick capillary blood sampling is preferred, as there is no need for a trained phlebotomist. However, the limited blood volume of fingerprick sampling requires alternative processing to separate blood into plasma. Various methods for automated point-of-care plasma separation of small volumes of blood have been investigated,^63^ however challenges remained with low separation efficiency, high dilution requirements, or clogging. Here we utilized microhematocrit capillaries and a miniature capillary centrifuge, which are commonly used to determine the hematocrit in clinical routine.

**Figure 6:**
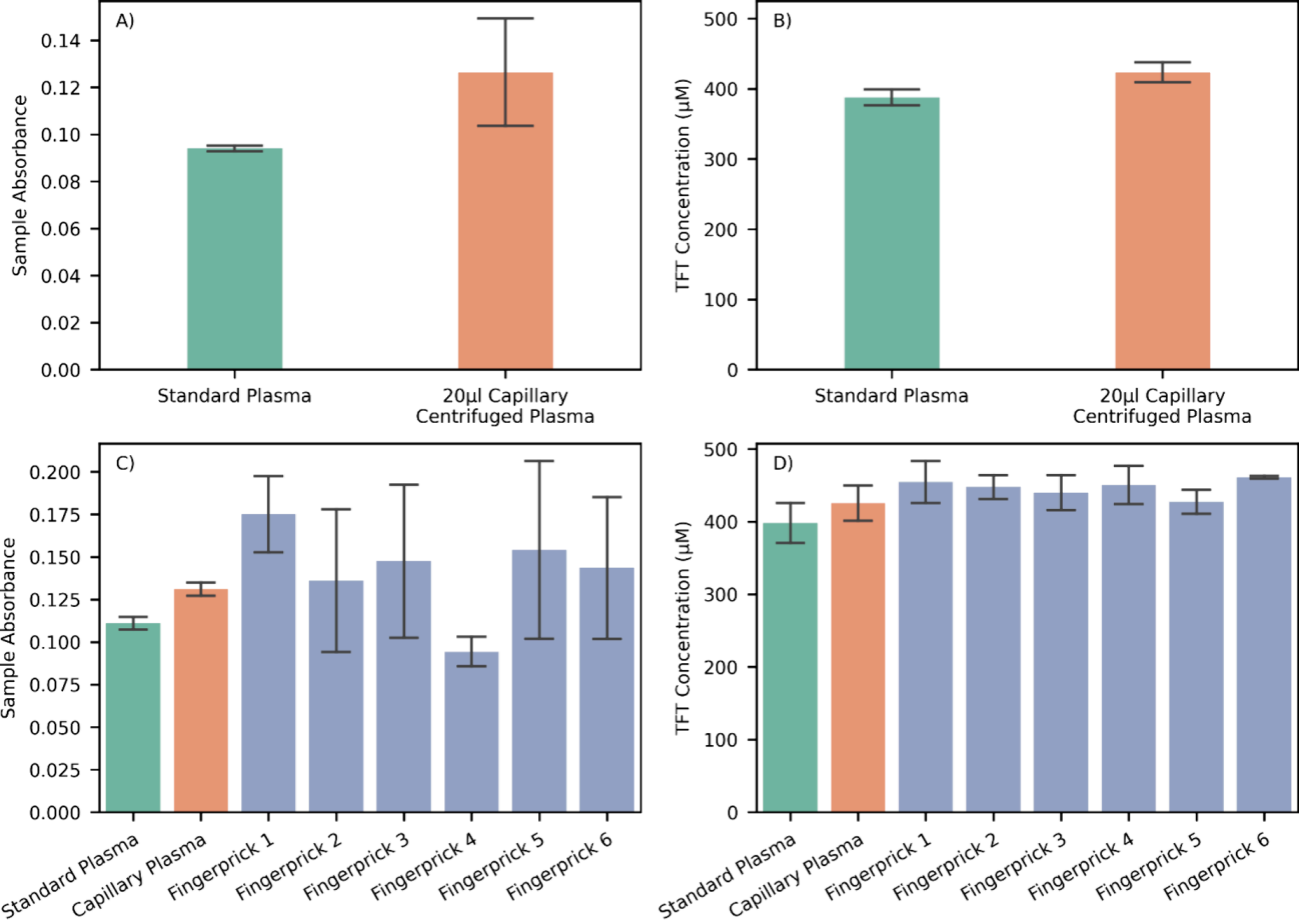
(A) Sample absorbance measured by the microfluidic system for plasma collected from a venous blood sample using a standard Eppendorf centrifuge and a capillary centrifuge with a 20 μL capillary. (B) Measured TFT concentration using the microfluidic system for the same blood sample and processing. (C) Sample absorbance measured using the microfluidic system for a venous blood sample centrifuged in commercial vacutainers or in capillaries, along with fingerprick capillary blood samples centrifuged in capillaries from the same participant. (D) Measured TFT concentration using the microfluidic system for the same blood sample and processing as shown in panel C. Bars show mean values of all repeats, and error bars show ±SD (*n* = 4).

It is common practice among clinical staff to fill these capillaries which reduces potential barriers to uptake; however, the plasma itself is not usually processed further. To check the suitability of using the plasma produced following centrifugation, a venous whole blood sample was loaded into 8 capillaries and centrifuged; the plasma collected from these capillaries was compared to the same venous sample processed in a standard Eppendorf centrifuge. The plasma collected from centrifuged capillaries shows clearly elevated background sample absorbance and slightly elevated TFT concentration compared with the reference plasma (Figure 6A,B). The elevated sample absorbance is indicative of a moderate degree of hemolysis occurring in capillary centrifuged samples. Free hemoglobin resulting from hemolysis of red blood cells shows three characteristic absorbance peaks with the largest at 414 nm, overlapping with the microfluidic system flow cells, and commonly used as an indicator of hemolysis.^64−68^ While absolute equality would be preferable, the consistent offset seen here will still enable trends and changes in the TFT concentration to be monitored as intended. In future versions of the system, the capillary centrifuge process could be fully automated.

### Fingerprick Blood Sampling

First, a venous blood sample was taken from a healthy volunteer and the plasma collected by centrifugation in the standard Eppendorf centrifuge and in 20 μL heparin-coated capillaries were analyzed by the microfluidic TFT system. A comparison fingerprick sample was taken from the same volunteer, and four 20 μL of heparin coated capillaries was filled from the blood flowing from the puncture site. These capillaries were centrifuged, and the plasma was processed by the microfluidic TFT system. After 3 h, a further five fingerpricks were conducted in the same participant, with between 2 and 4 capillaries filled from each puncture site, centrifuged, and analyzed. These fingerpricks were conducted over a relatively short time period with the participant not undergoing any stressors during this time that could be expected to induce oxidative stress and subsequent TFT changes. This test therefore acts as a comparison between venous and fingerprick-capillary blood TFT concentrations, along with the consistency within samples from a fingerprick and across multiple fingerpricks. The sample absorbance and TFT concentration recorded by the microfluidic system shows the same behavior for the venous sample seen previously, with the capillary centrifuged samples showing a moderately elevated sample absorbance and TFT concentration (Figure 6C,D), indicative of mild hemolysis. The fingerprick samples show a much greater variation in sample absorbance, both within and across punctures. Increased and variable hemolysis is a known factor in fingerprick blood samples compared with venous.^69−71^ The variable hemolysis level can be induced by a number of factors, including the donor, the puncture location as well as the free flow rate of blood, with an association of low puncture flow rate increasing hemolysis,^72^ a feature also noted in our testing. In cases where the absorbance of fingerprick samples was lower than that of the venous samples, we cannot exclude the potential contamination/dilution of capillary blood with interstitial fluid.^73^ Despite the variation in the measured sample absorbance, the calculated TFT concentration shows good consistency across the fingerprick samples (Figure 6D), showing that the correction for the sample absorbance is effective. The slight increase in TFT concentration observed in the fingerprick samples is again likely to be due to the increase in hemolysis.

### Total Free Thiol Monitoring

In order to demonstrate the ability of the microfluidic device to detect physiological changes in TFT concentrations, two healthy volunteers donated fingerprick capillary blood samples over the course of a normal day, which included potential physiological stressors. Sample collection and processing were as described above. The volunteers went about their normal day but reported when they ate or exercised. Participant 1 was a 30 year old male who was able to donate six samples on the morning of the test. The first of these samples was donated before the participant had breakfast and was therefore obtained in a fasted state. This participant subsequently donated two samples after breakfast and before lunch. The participant then exercised and had lunch before donating another 3 samples. The second participant, here identified as “participant 2”, was a 26 year old female who donated 3 samples in the afternoon. The first of these samples was taken shortly before the participant went to the gym, the second was taken shortly after the participant finished her workout, and the final sample was taken after a further hour of recovery.

**Figure 7:**
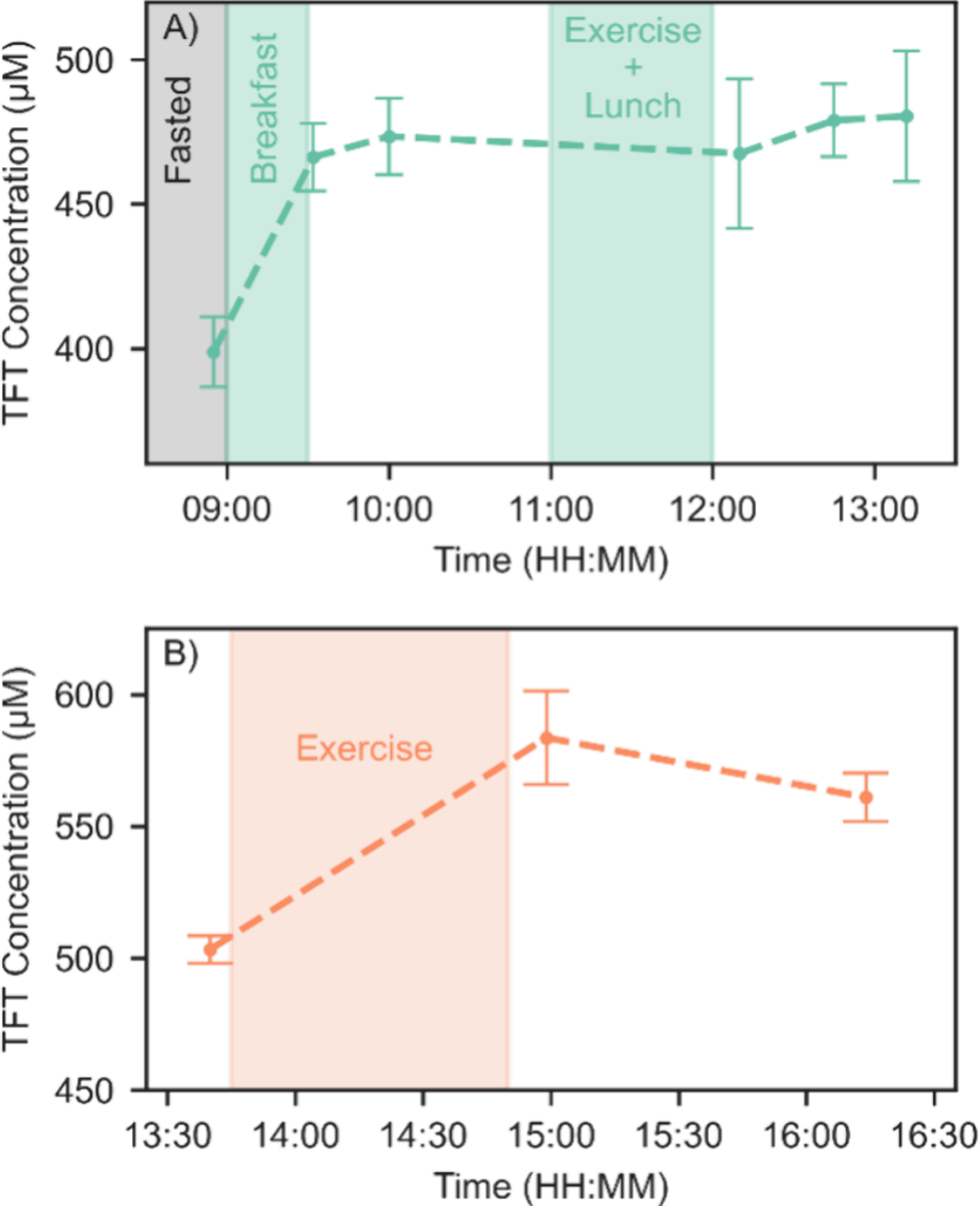
TFT concentration was measured in two participants using fingerprick sampling during a regular day with potential stressors highlighted. Points show the mean value recorded from each fingerprick, with four 20 μL capillaries collected from each fingerprick. Error bars show ±SD. (A) Participant 1. (B) Participant 2.

A clear increase in TFT is seen when participant 1 went from the fasted to the fed state (Figure 7A). Previously in a study of 10 healthy male volunteers, TFT has been shown to have a circadian rhythm with a minimum at 04:00, increasing slightly by 08:00 with a more significant increase of approximately 50 μM at noon, with breakfast having been consumed at 09:00.^74^ This behavior is similar to that seen in participant 1 of this study. Whether the nutritional intake after fasting is the cause of these changes or they result from an alternative circadian rhythm is unclear at this stage. For the remainder of the test, the TFT level of participant 1 remained the same despite further nutritional intake and exercise. By comparison, participant 2 experienced a marked increase in TFT concentration after exercising, followed by a decrease during recovery (Figure 7B). Previously, total antioxidant capacity, which would include free thiols, has shown an increase of 6.57–8.26% after moderate exercise,^75^ while reduced albumin, a specific free thiol, has been shown to decrease with exercise,^25^ but to our knowledge, no data are available for the response of total free thiols to exercise. Examining the previously reported circadian behavior shows TFT levels peaking at 16:00,^74^ and this effect may also be present in the samples of participant 2, as the timings are similar.

The difference in the TFT response between the two participants to exercise could be due to a number of factors including type, intensity, and duration of the exercise, individual physiology, variation in nutritional intake, and differences in exposome history and sex. Data on the effects of exercise and food intake on TFT levels are sparse, but since responses to both of these conditions are highly regulated by homeostatic conditions including neural, hormonal, and biochemical changes in redox state, variability in TFT concentration might be expected to occur.

## Conclusion

We here presented a portable point-of-care droplet microfluidic sensor capable of measuring TFT concentration in venous and fingerprick plasma samples. We have demonstrated that the sensor is robust to the variability induced by the fingerprick sampling method and shown that a small but consistent offset is observed between venous and fingerprick samples due to moderate hemolysis during the processing of capillary blood. We have demonstrated that the developed droplet microfluidic sensor can detect physiological changes in TFT concentration using fingerprick samples taken from healthy volunteers; showing changes potentially due to food intake and exercise. We, therefore, envisage this system as being capable of monitoring TFT changes using frequent fingerprick samples to identify previously unknown dynamic changes in redox status. By interfacing the device with other continuous sampling methods, such as microneedles or mirodialysis, the system could be used for the autonomous analysis and continuous monitoring of TFT levels and tracking their trajectory of change. The system could be further developed to allow multiplexing of multiple analytes, such as the total protein content or lactate, providing more information from the same blood sample. Ultimately, microfluidic sensors of this type may be used to gain new insight into the dynamics of redox alterations in physiology and provide a new window into whole-body redox balance; the latter will be of particular interest to the emerging field of redox medicine to provide biomarker-guided improvement to patient care in clinical settings associated with alterations in redox state.

## Supporting Information Available

Photos of prototype device and capillary processing, and calibration curves for converting a sample absorbance flow cell reading to an equivalent droplet absorbance flow cell reading (PDF)
