# Supplementary material for: A Droplet Microfluidic Sensor for Point-of-Care Measurement of Plasma/Serum Total Free Thiol Concentrations
Source: Anal Chem. 2025 Jan 27;97(5):2678–88. doi: 10.1021/acs.analchem.4c04163 (PMC11822743; doi:10.1021/acs.analchem.4c04163)
Supplement: Supplementary file 1 — ac4c04163_si_001.pdf [file ac4c04163_si_001.pdf]

Supporting Information

A Droplet Microfluidic Sensor for Point-of-Care Measurement of Plasma/Serum Total Free Thiol Concentrations

Liam Carter[1], Adrian Nightingale[1], Martin Feelisch[2,3*] and Xize Niu[1*]

[1]Mechanical Engineering, Faculty of Engineering and Physical Sciences, University of Southampton, Southampton, UK. [2]Perioperative and Critical Care Theme, NIHR Southampton Biomedical Research Centre, University Hospital Southampton, Southampton, UK. 3 Clinical & Experimental Sciences, Faculty of Medicine, University of Southampton, Southampton, UK

Contents

List of figures

Figure S1 Prototype device …………………………………………………………………….……….S2

Figure S2 Photo of capillary processing ……………………………………………………..….S2

Figure S3 Calibration curve for converting a sample absorbance flow cell reading to an equivalent droplet absorbance flow cell reading. ………………………………………………………………..S2

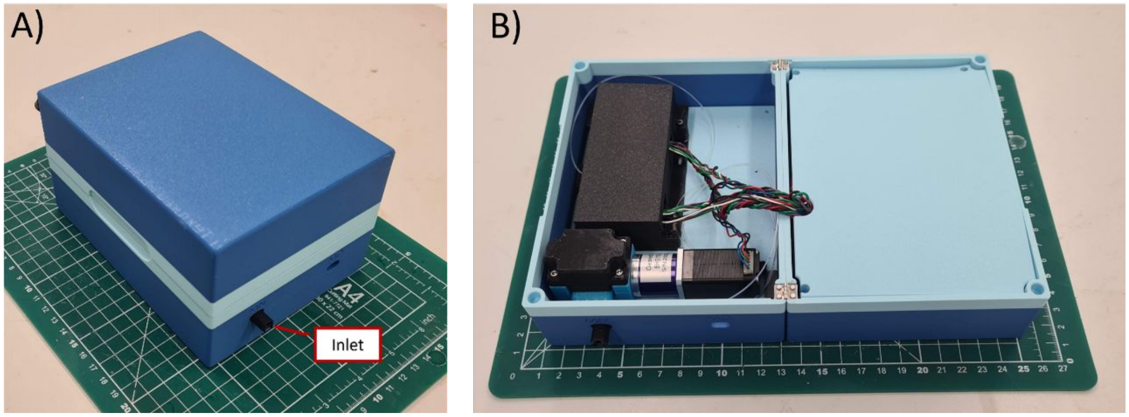

Figure S1: Prototype device. A) Photo of the fully assembled and integrated device, showing the inlet for capillaries. B) Open device showing pump and fluidics (contained within the black box) on the left and control electronics enclosed on the right.

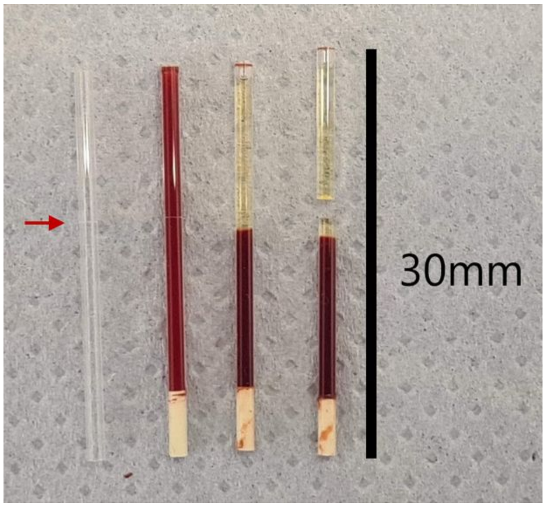

Figure S2: Photo of capillary processing, left to right: Empty capillary with score mark indicated (1), capillary filled with blood and wax plugged (2), centrifuged capillary showing separation of blood (3), snapped capillary with plasma region isolated ready for loading (4).

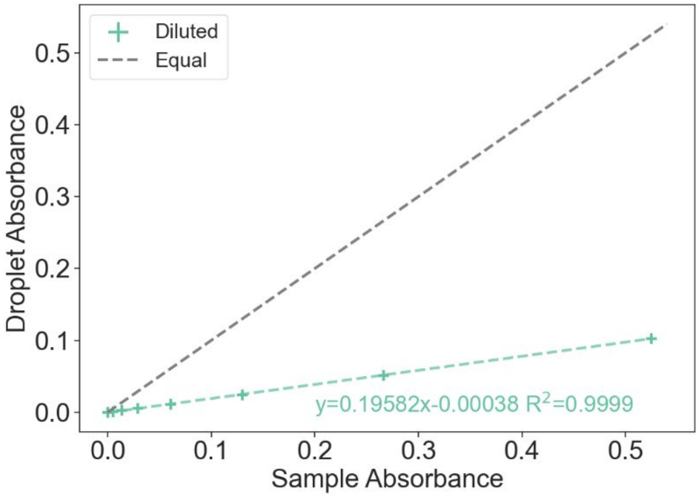

Figure S3: Calibration curve for converting a sample absorbance flow cell reading to an equivalent droplet absorbance flow cell reading. Accounting for dilution and performance variation between flow cells. Calibration was measured using a range of concentration solutions made from yellow food dye (PME Paste, which shows a similar absorbance spectrum to the plasma background and reaction product) as samples and MQ water in the reagent and buffer lines.
